# 

**Published:** 1901-06

**Authors:** 


South Texas Medical Association.

### South Texas Medical Association.

HOUSTON, TEXAS, May 6, 1901.

DEAR DOCTOR: You are earnestly requested to furnish a paper for the next meeting of the South Texas Medical Association, which occurs in Houston, June 20th and 21st. Please send title of your paper on or before June 1st.

Fraternally yours,

R. W. KNOX, President,

Houston.

D. S. WIER, Secretary,

Houston.

